# Back to Basics: Recognition of Sepsis with New Definition

**DOI:** 10.3390/jcm8111838

**Published:** 2019-11-01

**Authors:** Jan Horak, Vendula Martinkova, Jaroslav Radej, Martin Matejovič

**Affiliations:** 1 Department of Internal Medicine, Faculty of Medicine in Pilsen, Pilsen University Hospital, Charles University Prague, Alej Svobody 80, 323 00 Pilsen, Czech Republic; HORAKJAN@fnplzen.cz (J.H.); radejj@fnplze.cz (J.R.); 2 Biomedical Centre, Faculty of Medicine in Pilsen, Charles University Prague, Alej Svobody 80, 323 00 Pilsen, Czech Republic; martinkova.vendula@gmail.com; 3 Third Department of Surgery, University Hospital Motol and First Medical School, Charles University, V Uvalu 84, 150 06 Prague, Czech Republic

**Keywords:** infection, sepsis, septic shock, early recognition of sepsis, definition, risk assessment

## Abstract

Patients with serious infections at risk of deterioration represent highly challenging clinical situations, and in particular for junior doctors. A comprehensive clinical examination that integrates the assessment of vital signs, hemodynamics, and peripheral perfusion into clinical decision making is key to responding promptly and effectively to evolving acute medical illnesses, such as sepsis or septic shock. Against this background, the new concept of sepsis definition may provide a useful link between junior doctors and consultant decision making. The purpose of this article is to introduce the updated definition of sepsis and suggest its practical implications, with particular emphasis on integrative clinical assessment, allowing for the rapid identification of patients who are at risk of further deterioration.

## 1. Introduction

Sepsis is the primary cause of death from infection. It affects 30 million patients annually, of which 25%–30% die [1]. This fact is reflected in recent data from the Czech multicentric project EPOSS (Data-based Evaluation and Prediction of Outcome in Severe Sepsis), which points to a 40% in-hospital mortality of patients diagnosed with severe sepsis [2]. A patient admitted with severe sepsis runs a 6–10 × higher risk of death than a patient admitted with acute myocardial infarction and a 4–5 × higher risk than the patient admitted with stroke. There are only few disease processes with such a high mortality. Qualified estimates and epidemiological data today clearly rank sepsis as one of the most frequent causes of death in general. In developed countries, the number of patients hospitalised with sepsis has doubled over the past eight years and is higher than the number of patients hospitalised with myocardial infarction. Sepsis is the cause of 30%–50% of all in-hospital deaths [3]. Infections complicated by sepsis also have a significantly negative impact on the results of all medical specialities. Awareness of the long-term consequences of sepsis is on the rise. Patients who survive sepsis suffer from long-term physical, mental, and cognitive disorders that have a significant health, social, and economic impact, and they remain at increased risk of death many years after the episode of sepsis [4]. The financial costs of treating sepsis in the inpatient setting expended by every healthcare system are enormous, reaching 5.2% of all healthcare costs in developed countries [5]. Despite these alarming figures, awareness of sepsis by both the lay and professional public remains overshadowed by cardiovascular or oncological diseases. In this context, early recognition of sepsis and risk assessment of patients with serious infections remains a fundamental challenge in clinical practice. The purpose of this article is to introduce the updated definition of sepsis, discuss its limitations on the background of available evidence and suggest its practical implications, with particular emphasis on integrative clinical assessment allowing for rapid identification of patients who are at risk of further deterioration.

## 2. Does This Patient Have Sepsis?

### Clinical Vignette

*A 19-year old man wounded in the thigh when playing sports. After 24 h, he is brought to the outpatients department by his family, suffering from pain in the wounded limb, repeated vomiting, fever, tachycardia, and general exhaustion. A 77-year old patient with a history of chronic renal disease is brought to the emergency department suffering from confusion, dyspnoea and abdominal pain arising over a period of several hours. A 58-year old woman is waiting to be seen by the neurologist. She has a two-day history of limb weakness and general malaise, and she suffers an episode of syncope in the waiting room*. Three patients, three fates, with different complaints, but the same diagnosis: Sepsis.

Recognition of sepsis itself has changed markedly over time. Hippocrates was the first to use the term *sipsi* (from the Greek *make rotten*) as early as the 4th century BC. However, sepsis became associated with infection only thanks to the discoveries of L. Pasteur [6]. The first definition of sepsis arose only much later, when in 1989 Dr. Bone presented sepsis to the professional public as a state induced by the invasion of microorganisms and/or their toxins into the bloodstream, along with the organism´s reaction against this invasion [7]. A few years later, this definition was replaced by the concept of the systemic inflammatory response syndrome (SIRS), which in combination with the confirmed or merely presumed presence of infection defined sepsis. Severe sepsis then referred to a condition involving one or more organ dysfunction. The last category within the classification of septic states was septic shock, defined in 1991 as sepsis complicated by hypotension refractory to fluid resuscitation [8]. In 2001, an extensive list of other signs and symptoms was added to the criteria of sepsis. The definition of sepsis based on the concomitant presence of infection and SIRS criteria was repeatedly criticised for its insufficient sensitivity and specificity and its disproportionate emphasis on the concept of excessive inflammatory response. This criticism was also recently supported by results documenting that one out of eight patients diagnosed with sepsis and concurrent acute organ dysfunction does not meet even two of the four SIRS criteria [9]. Similarly, a considerable percentage of patients with a physiological, i.e., desired response of the immune system to a non-serious infection meet these criteria quite easily, although they are not suffering from sepsis [10].

In 2016, The Journal of American Medical Association published a new definition of sepsis, referred to as SEPSIS-3 [11]. This definition was formulated on the basis of the retrospective analysis of data from 1.2 million patients with infection. The aim of this analysis was to uncover data that would be able to increase the accuracy and rapidity of diagnosing sepsis, thus enabling the differentiation between an uncomplicated infection and one that leads to acute organ dysfunction, and that is associated with a significantly higher risk of death. Sepsis is redefined as life-threatening organ dysfunction caused by a dysregulated host response to infection. This definition thus requires the identification of acute organ dysfunction. The Sequential/Sepsis-related Organ Failure Assessment (SOFA) score has been devised to this end [12]. Using a scale (0–4 points), it evaluates respiratory function, coagulation parameters, liver and renal function, haemodynamic parameters, and level of consciousness. The SOFA score requires a number of laboratory tests that may not always be available in time. With the aim of simplifying and accelerating assessment for the presence of sepsis induced organ dysfunction in the pre-admission phase, in emergency departments as well as on general wards, the SEPSIS-3 working group recommends the use of the so-called qSOFA (quickSOFA) score. This is a system based on three criteria (points), where each represents a deviation in a single vital parameter: low blood pressure (systolic blood pressure, BPs ≤ 100 mmHg), tachypnoea (≥22 breaths per minute), and altered mental status (Glasgow Coma Scale < 15). The presence of two or more qSOFA criteria in a patient suffering from infection indicates a high probability of sepsis, and a significantly higher risk of an unfavourable course of the acute illness. Septic shock now refers to a state when sepsis is complicated by hypotension refractory to fluid resuscitation, and which requires vasopressor administration to maintain mean arterial pressure ≥ 65 mmHg, and a concurrent blood lactate level higher than 2 mmol/L. This condition is associated with a more than 40% in-hospital mortality [11]. The proposed definition of sepsis will undoubtedly have its opponents and proponents [13,14]. The critics will argue that the imperfect original definition has been replaced by an equally imperfect definition that has not been verified by robust prospective data. Objections may be raised that waiting for signs of organ dysfunction may paradoxically delay the diagnosis of sepsis, or rather, the timely institution of therapeutic measures (the same argument applies to the SIRS based concept) [13,14]. The SOFA score is not universally applied in clinical practice and is more of a predictor of mortality than a diagnostic test, and surely may not be uniquely interpreted as a tool for sepsis screening. The new definition is not going to be quite as clinically useful in the intensive care setting, as the basic components of qSOFA are usually modified by therapeutic interventions (mechanical ventilation, analgosedation) and the deterioration in a patient´s condition as a consequence of sepsis occurs without any concomitant changes in the qSOFA. On the other hand, proponents of the new approach find that the new definition represents a conceptual shift in the perception of the pathobiology of sepsis—as many conditions that are currently classified as sepsis are simple infections associated with an adaptive systemic inflammatory response that does not lead to organ dysfunction. The original distinction between sepsis and severe sepsis no longer has any pathobiological rationale. On the contrary, the initial term severe sepsis corresponds to the proposed definition of sepsis in the light of current findings. The proposed concept will help clinicians in triage with patients suffering from infection, as it will improve the perception of sepsis as a true “medical emergency“, i.e., a state meriting immediate diagnostic and therapeutic management, including the deliberation of where the given patient should proceed to (ICU, versus the general ward). The advantage of the qSOFA is its non-invasiveness, speed, and the ability to repeatedly re-assess the patient´s condition directly at the bedside in a general ward or outpatient unit.

## 3. Implementation of the New Definition in Clinical Practice

The publication of SEPSIS-3 provoked a fierce discussion, and the new definition has not by far been officially accepted by all professional associations [15]. It can be presumed that the proposed concept will not represent a fundamental change in clinical decision making and processes for the experienced physician. Early recognition of sepsis and assessment of its seriousness remains for the experienced clinician a case of subconsciously applying the Bayesian modelling-hypothesis that H applies, i.e., the patient probably has sepsis if we observe evidence E, i.e., the complex of clinical and complementary data. Such an approach includes the simultaneous evaluation of a number of basic clinical information factors: the vital functions, the presence and degree of systemic inflammatory response (biomarkers of inflammation and infection, e.g., a CRP of 500 mg/L might carry a different weight compared to a CRP of 50 mg/L), and the careful assessment of hemodynamic parameters, multiple organ functions, and tissue perfusion (see below). However, we believe that SEPSIS-3 provides a clinically useful conceptual framework mainly for less experienced physicians, outpatient departments, pre-hospital care, or general wards. Indeed, the simple definition and criteria of sepsis and septic shock stress three minimum steps in the clinical assessment and management of a patient: (1) determining the diagnosis of infection and its timely treatment (which includes samples for microbiological testing including blood cultures, and the timely administration of adequate antibiotics); (2) active assessment of acute organ dysfunction for the rapid stratification of patient risk; (3) recognition and treatment of hypotension as one of the key prognostic factors (i.e., administration of balanced crystalloid fluids and the immediate assessment of their effect, consultation of intensivists). Naturally, it is necessary to keep in mind that the new definition does not represent a change in recommended therapeutic procedures.

Thanks to its heterogeneity, sepsis will remain a diagnosis that is difficult to make, as no gold standard exists to verify this diagnosis. The high variability and degree of subjectivity when determining the diagnosis of sepsis is clearly documented by a recent survey of 94 intensivists from academic centres, which showed significant inter-individual differences when evaluating five fictitious yet clinically quite realistic cases of sepsis [16]. We will continue to see a population of patients who will fail to meet any of the definitions of sepsis, despite suffering from this syndrome. These will predominantly include patients in whom sepsis is a complication of another (or other) serious acute or chronic condition(s). Similarly, with the absence of SIRS, a negative qSOFA does not rule out the presence of sepsis. On the contrary, its positivity is not an absolute synonym for sepsis, despite the concurrent presence of infection (e.g., medication induced alteration of consciousness in the peri-operative period; chronic cognitive deficit; tachypnoea induced by anxiety, pain, or chronic obstructive pulmonary disease exacerbation). The axiom in clinical practice remains that if a well-founded clinical suspicion of sepsis arises (the patient “looks ill” or “deteriorates suddenly and unexpectedly“, and we do not have any other explanation for this), we must always proceed regardless of whether diagnostic criteria have been met or not. Thus, the new concept of defining sepsis is not immune to various limitations, and it awaits a prospective validation and further adjustments. Although two studies demonstrated consistent findings to those of the Sepsis-3 taskforce (thus supporting external validity for the new definition [17,18]), its robustness as well as its discriminatory ability has recently been questioned [19,20]. Similarly, the prognostic and diagnostic accuracy of qSOFA remains a contentious issue. Most recent retrospective studies [21,22,23,24,25,26,27,28,29] validating the sensitivity, specificity, and predictive values of qSOFA (Table 1) suggest that qSOFA may not be as robust as originally thought. In addition, recent systematic reviews and meta-analyses showed that a positive qSOFA score has high specificity, but low sensitivity outside the ICU in early detection of in-hospital mortality, acute organ dysfunction, and ICU admission [30], and a greater prognostic accuracy for in-hospital mortality as compared to SIRS [31,32]. By contrast, other studies reported poor sensitivity and moderate specificity for predicting mortality [33,34]. It must be stressed, however, that most studies evaluated prognostic accuracy of qSOFA for mortality, rather than diagnostic accuracy, thus limiting calculations concerning predictive values [35]. In addition, the small number of prospective studies included in the meta-analyses largely limits the validity and generalizability of the results. Nevertheless, it appears that the SIRS criteria are more sensitive for diagnosing sepsis, while the qSOFA criteria are more accurate for predicting in-hospital mortality among patients identified with sepsis [34]. This reasoning is supported by Serafim et al., who suggested in their systematic review that an association of both qSOFA and SIRS criteria could provide a better model to initiate or escalate therapy in patients with sepsis [35]. Taken together, because data of the value of qSOFA remains conflicting, further studies that demonstrate improved clinically meaningful outcomes due to the use of qSOFA are warranted. Nevertheless, despite all the above mentioned uncertainties, we believe that the new concept may change (in the positive sense of the word) current perceptions of sepsis, including the way in which this fundamental issue is conveyed to the professional and lay public.

## 4. Sepsis in the Emergency and General Ward Setting—Back to Basics

### Clinical Case

*A 72-year old patient is being examined at the emergency department for a community-acquired pneumonia. The physician from the intensive care unit is called in for a consultation regarding the patient´s admission to a general ward or the ICU. The physician is confronted with a mildly confused patient wearing an oxygen mask thanks to which his haemoglobin oxygen saturation is 94% (without the mask it is 86%). He finds that the patient´s pulse is irregular, 110–135/min; the urinary catheter contains concentrated dark urine, and the urinary drainage bag is nearly empty after two hours of examinations at the admissions department. The blood pressure is between 95–105/55–65 mmHg following the administration of 1 litre of a crystalloid fluid. What will the decision be?*

Infectious diseases in the USA are responsible for more than 10 million visits to emergency departments annually, and similar epidemiological data apply in Europe. In the population of patients over the age of 65 years, infections are a more frequent cause of hospitalization than heart failure and myocardial infarction all together [36]. Of these cases, a substantial number (36%–54%) are admitted to general wards [37], whereby one in five of such patients develop septic shock within the subsequent 72 h [38]. The Czech EPOSS project showed that the prognosis of septic patients referred to ICUs from general wards is significantly worse (45.1%) than in the case of patients admitted primarily from low-threshold departments (26.5%) [2]. Sepsis is also the most frequent cause of acute deterioration in patients on general wards. The emergency departments of hospitals, as well as general wards, are thus frequently the sites of the initial evaluation of septic patients. Clearly, the challenge is to recognise those patients at great risk of progression or death, especially patients who are not initially critically ill.

As mentioned above, the absence of one of the screening criteria of sepsis (qSOFA) does not rule out the risk of progression of sepsis to septic shock or multiorgan dysfunction. Careful clinical examination (head to toe) remains an essential tool for evaluating alert signs and symptoms that indicate a more serious underlying pathology (red flag symptoms). Peripheral tissues, e.g., skin, are among the first to show clinical signs of altered hemodynamics and tissue perfusion in severe infections [39,40,41]. Three simple, non-invasive, and in every situation evaluable warning signs include: (1) The presence of skin mottling (patchy skin discolouration) typically manifested in the knee area, fingers, or toes, and reflecting altered microcirculation. A simple score from 0 to 5 according to the size of the affected area from the knees down to the periphery closely correlates with mortality, independent of systemic hemodynamics [42]. A high score after six hours from the initial haemodynamic resuscitation is a strong predictor of 14-day mortality [42]. (2) Delayed capillary refill time, i.e., the time taken for blood flow to return to the nail bed following compression for 5 s. Delayed refill (there is no uniformly validated time definition, and cut-off is set at 2.5–4.5 s) in the general population of critically ill patients correlates with the severity of organ dysfunction, predicts mortality, and on the contrary, its normalization is associated with better survival [42,43,44]. (3) The temperature gradient between the forearm and fingers (cool acral parts), assessed both subjectively or measured exactly. A difference greater than 4 °C is a sign of significant peripheral vasoconstriction and poor peripheral tissue perfusion [44].

Apart from the assessment of vital functions, hemodynamics, and peripheral perfusion, the third useful step is the evaluation of blood lactate levels. In septic patients, lactate is as important as the highly sensitive troponin in acute coronary syndromes. It is a key marker of metabolic cell stress. Even a slightly increased level of lactate (2.1–3.9 mmol/L) is associated with higher mortality, whereby the risk of death increases precipitously at lactate levels above 4 mmol/L. Testing lactate levels is important not only in hypotensive patients where increased levels—despite use of initial fluid resuscitation—confirm the development of septic shock (“overt shock“). Its determination is of exceptional benefit in another sub-group of septic patients at high risk of an unfavourable course, namely in so-called occult or cryptic shock. This is a phenotype of sepsis characterized by normal blood pressure, which may lack any other signs of acute organ dysfunction or clinical manifestations of shock. We must remember that the septic patient may be “more ill” than he or she appears to be at the given time. This situation arises in nearly one half of patients who do not have a BPs < 90 mmHg [45]. Increased lactate levels in otherwise normotensive patients together with hyperthermia represent strong predictors of early (within 72 h) development of septic shock despite effective and timely antibiotic treatment [38,46]. This sub-group of patients requires very careful evaluation, preferably in cooperation with an intensivist. If for any (well documented) reason, we decide to admit such a patient to a general ward, it is necessary to ensure his/her close monitoring and frequent re-evaluation of the effect of the treatment provided. Contrary to common belief, venous blood is useful for determining lactate levels. It is handy to collect the sample for lactate analysis first, once the tourniquet is applied to the extremity, but in general short constriction does not affect the result. In view of the accessibility of point-of-care testing blood gas analysers in emergency departments, the result is available within a few minutes. Another sepsis phenotype represents a group of patients with persistent hypotension and completely normal lactate levels. It would be a mistake, however, to conclude based on the information detailed above that this is not an emergency situation requiring treatment in an intensive care unit. While this group of patients has a better prognosis, the risk of an unfavourable disease course still exists.

An isolated evaluation of any clinical or laboratory indicator is not a sufficiently sensitive or specific tool for definitely assessing tissue perfusion. In the multimodal concept however, these represent simple, easily repeated, and significant warning signs that enable, in most cases, the rapid triage of any patient whose condition deteriorates suddenly. Similarly, the evolution of these indicators following initial intervention is an important signal of the efficacy of therapeutic measures. Nonetheless, even a patient whose warning signs resolve in response to initial therapeutic management deserves frequent re-evaluation in the first 48 h of hospitalization.

The patient described above ranks among at-risk patients (encephalopathy, hypoxemia, relative hypotension, two hours of anuria, tachyarrhythmia). Further information must be gained and his response to subsequent therapeutic measures must be carefully evaluated. These measures include continuing with fluid administration (the initial dose of balanced crystalloid fluids in patients with no signs of fluid overload is 20–30 mL/kg), evaluation and potential correction of fever (hyperthermia may play a role in the altered mental status, tachypnoea, atrial tachyfibrillation, but CAVE: risk of hypotension following the administration of paracetamol or metamizole), and early and appropriate antimicrobial therapy with the sampling of microbiological material including blood cultures. A multimodal evaluation of clinical signs of hypoperfusion, lactate levels, acid-base balance, and laboratory signs of other organ dysfunctions takes place in parallel. Improvement of the encephalopathy, slowing of the heart rate, absence or correction of the aforementioned skin abnormalities, restoration of diuresis, and physiological levels of lactate may justify further treatment in a general ward. In the opposite case, the patient should be transferred to an intensive care unit, unless the context dictates otherwise (e.g., the terminal stage of a chronic disease).

## 5. Conclusions

Sepsis, the primary cause of death from infection, is newly defined as a life-threatening organ dysfunction caused by a dysregulated host response to infection. Sepsis must be perceived as a “medical emergency”, i.e., a condition that requires immediate diagnostic and therapeutic management, including a timely decision as to where the given patient should be heading next. Evaluation of the qSOFA together with clinical signs of peripheral hypoperfusion and determination of blood lactate levels represent the basic, initial tools for the safe triage of patients suspected of having sepsis. However, a well-founded clinical suspicion of sepsis (the patient “looks ill” or “deteriorates suddenly and unexpectedly”, and we have no other explanation for this) entitles us to take active steps regardless of whether the criteria of the definition have been met or not. The three basic steps include: (1) determining the diagnosis of infection, including its timely treatment; (2) assessment of acute organ dysfunction in order to rapidly stratify the patient’s risk; (3) recognizing and immediately treating hypotension. A patient in whom the aforementioned “red flags” are present requires activation of the process/system: admitting physician → intensivist, general ward physician → intensivist.

## Figures and Tables

**Table 1:** Recent studies validating the sensitivity, specificity, and prognostic values of qSOFA scores. PPV: Positive predictive value; NPV: Negative predictive value; ED: Emergency department.

Study	Patients	End-Point(s)	qSOFA	Sensitivity (%)	Specificity (%)	PPV (%)	NPV (%)
***Probst et al. (2019)*** [21]	Retrospective cohort N = 450 (Hematological-cancer)	Diagnostics Hospital mortality (28-day mortality)	≥2	41.5 (34.2–49.1) 45 (35.6–54.8)	91.1 (86.8–94.0) 85.5 (81.1–89.0)	- -	- -
***Tian et al. (2019)*** [22]	Retrospective cohort n = 1716 (Emergency department)	Diagnostics	≥2	50.2	78.1	73.3	56.7
***Lane et al. (2019)*** [23]	Restrospective n = 10409	Hospital mortality	1 2 3	80 (77–83) 37 (34–40) 8 (7–10)	53 (51–53) 91 (91–92) 99 (99–99)	14 (13–15) 29 (26–32) 53 (44–61)	96 (96–97) 94 (93–94) 92 (91–92)
***Liu et al. (2019)*** [24]	Retrospective cohort n = 1865 (Sepsis, septic shock)	30-day mortality 90-day mortality 1-year mortality	≥2	23.6 23.0 23.2	82.2 82.7 83.8	49.6 59.4 67.3	41.6 50.9 56.8
***Harada et al. (2019)*** [25]	Restrospective, single-site n = 4827 (Pre-hospital, emerg dpt.)	Hospital mortality	≥2	52.3	69.9	-	-
***Luo et al. (2019)*** [26]	Retrospective, single-site n = 409 (Hospitalized, gen. ward)	Diagnostics	max	53 (47–60)	87 (81–91)	84 (77–89)	59 (53–65)
***Usman et al. (2018)*** [27]	Retrospective n = 930 (Sepsis, septic shock)	Diagnostics Hospital mortality	1 2 3 1 2 3	71.8 28.5 5.4 83.3 43.3 10.7	87.2 98.9 99.9 86.7 98.7 99.9	- - - - - -	- - - - - -
***Shu et al. (2018)*** [28]	Retrospective observational n = 2292 (Pre-hospital)	Diagnostics In-hospital mortality	≥2	42.9 (35.1–51.0) 40.6 (25.3–58.1)	93.8 (92.6–96.8) 6.6 (3.9–11.0)	-	-
***Gaini et al. (2019)*** [29]	Retrospective n = 323 (Emergency dpt.)	Hospital mortality	≥2	38.1 (18.1–61.6)	89.1 (85.0–92.4)	-	-
